# Evaluating complex health interventions: a critical analysis of the 'outcomes' concept

**DOI:** 10.1186/1472-6882-9-18

**Published:** 2009-06-18

**Authors:** Charlotte Paterson, Charlotte Baarts, Laila Launsø, Marja J Verhoef

**Affiliations:** 1 Institute of Health Services Research, Peninsula Medical School, University of Exeter, Exeter, UK; 2 Department of Sociology, University of Copenhagen, Øster Farimagsgade 5, POB 2099, DK-1014 Copenhagen K, Denmark; 3 The National Research Center in Complementary and Alternative Medicine (NAFKAM), The Medical Faculty, University of Tromsø, 9037 Tromsø, Norway; 4 The Danish Multiple Sclerosis Society, Mosedalvej 15, 2500 Valby, Denmark; 5 Department of Community Health Sciences, University of Calgary, 3330 Hospital Drive NW, Calgary, T2N 4N1, Canada

## Abstract

**Background:**

The extent to which a health care intervention causes or facilitates health-related change is a key question in research. The need to quantify such change has led to the development of an increasing number of change indicators, to measure what have come to be known as 'outcomes'. In the context of medical research into the efficacy or effectiveness of an intervention the term 'outcomes' has often been interpreted to mean single endpoints with a linear cause and effect link to an external intervention.

**Discussion:**

In this paper we present a critical analysis of the nature and interpretation of the 'outcomes' concept and of the assumptions that underpin it. Drawing on our own work and that of others, we analyse the problems that arise when the concept is applied to complex interventions and discuss the use of other models, such as programme theory, as a basis for alternative conceptualisations for indicators of change.

Our analysis demonstrates that the interpretation of 'outcomes' that may be appropriate for clinical trials of pharmaceutical products, is problematic when used in evaluations of complex interventions in areas such as complementary medicine, palliative care, rehabilitation, and health promotion. The 'outcomes' concept may impose inappropriate patterns of thought and meaning. We present alternative models, such as those based on programme theory, which conceptualise health-related change as resulting from the interaction between intervention, process and context over time. In this framework both the intervention and the patient are defined as causal factors, because the result of the treatment is dependent on the resources of the patient – such as the body's ability to heal itself – and the impact of the patient's situation.

**Summary:**

Evaluations based on a model such as programme theory will encompass a wide range of health-related changes that include aspects of process, such as new meanings and understanding, as well as longer term changes in health, wellbeing and health-related competences and behaviours.

## Background

**Figure 1:**
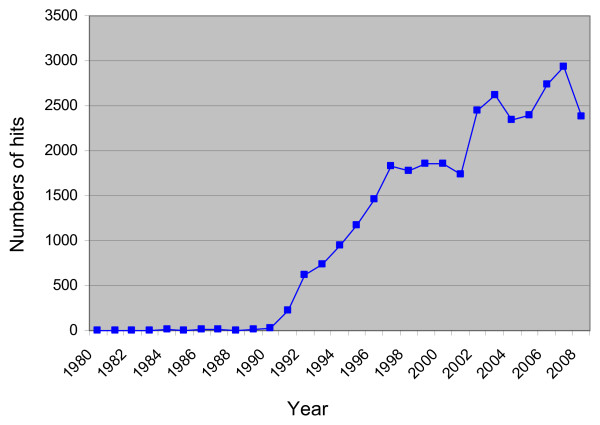
**Number of 'hits' on Medline (on Ovid) search for "outcome assessment (health care)" or "outcome assessment".mp**, Limited by year.

The extent to which a health care intervention causes or facilitates health-related change is a key question in research into complementary and alternative medicine. It is a question posed by patients, providers and policy makers and is researched at different levels using a range of research designs and qualitative and quantitative methods. The need to quantify such change has led to the development of an increasing number of change indicators, which include 'objective' and 'subjective' parameters. These indicators have come to be known as 'outcomes' and measuring these outcomes, by means of validated instruments and questionnaires, is now an important field of academic activity that generates a plethora of health-related outcome measures and associated methodological debate. Figure 1 illustrates the exponential increase in academic activity related to this contemporary conceptualisation of 'outcomes' over the last twenty years. In relation to complementary and alternative therapies, this activity includes both generic and problem-specific measures and their use in randomised controlled trials (RCTs) and descriptive outcome studies [1-3]. However, in the context of medical research into the efficacy or effectiveness of an intervention, there has been limited critical analysis of the nature and interpretation of the 'outcomes' concept or of the assumptions that underpin it. As outcomes assessment is essential in determining whether and how health-related change occurs, such an analysis is urgently needed.

Biomedicine has been highly successful in assessing the outcomes of pharmaceutical interventions on the basis of changes in pathology, symptoms, and other biological indicators. This assessment is usually focused on a single organ system or disease process. However, the application of these outcome measures to interventions in areas such as complementary medicine, palliative care, rehabilitation, mental health and health promotion is proving to be problematic. In these situations, people often experience a wide range of changes that extend beyond the common biomedical, psychological or quality of life outcomes and they may emphasize the process of healing as well as the impact of contextual factors [4-13]. The interrelated nature of treatment processes and outcomes and the variable and often long timeframe of change are becoming recurrent themes in research papers in these areas [14-18]. In addition, the alternative therapeutic and healing theories that underpin many of these interventions have important implications for the outcomes that are aimed for and experienced [19-22]. While there has been considerable debate about overall research design for complex interventions, especially the use of randomised controlled trials (RCTs) [20,23-29] it is not our intention to focus on this debate here. In this paper we focus on the meaning of the concepts 'outcomes' and 'outcome measurement', which are core concerns for both observational and experimental research and programme evaluations.

In the context of medical research into the efficacy or effectiveness of an intervention, where the RCT remains top of the hierarchy of evidence, the term 'outcomes' is generally interpreted to mean single endpoints with a linear cause and effect link to an external intervention. Whilst this may be appropriate in the discourse in which the term originated, clinical trials of pharmaceutical products, the continuing use of the term in other discourses may impose inappropriate patterns of thought and meaning. Take, for example, the use of 'outcomes' to describe changes in health, wellbeing and health-related behaviours in the context of someone with long-term illness engaging in a complex intervention – such as someone with arthritis and depression engaging in group exercise such as Tai Chi. In this situation, this interpretation of 'outcomes' may narrow the assessment of change to a single quantifiable indicator and divert attention away from the important contextual factors, feedback loops, the users' experiences and learning processes and individualised long-term outcomes. Calls for the use of a wider range of patient-centred outcome measures, especially in areas such as rehabilitation, mental health [18], and complementary medicine [30] have had only a limited effect on outcome measurement in clinical trials. This interpretation of the concept of 'outcomes' tends to have the effect of 'freezing' experiences at one point *in *time, although experiences change *over *time, and it does not illuminate the potential and limits of the health-related changes. Our own research into patients' experiences of complex interventions leads us to believe that a critical analysis of the concept of outcomes will assist in identifying and better understanding the challenges that we all face, in whatever discipline, in measuring outcomes appropriately.

This paper will trace the evolution of the concept of 'outcomes' from its beginnings in clinical trials of pharmaceuticals to its current use in evaluations of complex interventions. In so doing we will examine the assumptions that underpin the use of the concept in pharmaceutical trials and how they play out when it is used in different circumstances. Drawing on our own work and that of others, we will highlight the problems that arise when the concept is applied to complex interventions and use the example of programme theory to illustrate how researchers can develop alternative conceptualisations for indicators of change. Finally we will return to the outcomes of clinical trials of pharmaceuticals and reflect on the current problems in putting evidence from these trials into practice and how a new understanding of outcomes can help to overcome them.

## Discussion

### A. Critical Analysis of the Concept of 'outcomes'

#### Outcomes in a disease-focused pharmaceutical trial

Starting with the development of the concept of 'outcome measurement' in pharmaceutical trials, let us consider a placebo controlled trial of a drug which aims to counteract, at a molecular level, the inflammatory response in rheumatoid arthritis (RA). The drug is known experimentally to have its maximal effect after 8 weeks and so the primary outcome chosen is an objective measure of the degree and number of swollen joints at 8 weeks. The researchers, following best practice, include as secondary outcome measures the core set from the OMERACT (Outcome Measures in Rheumatoid Arthritis Clinical Trials) conference – often held up as an example of excellence in outcome measurement- plus some patient-centred subjective measures as recommended by more recent OMERACT workshops (See Table 1 for details of OMERACT). Within this context we can see that the concept of outcomes, as an 'endpoint', reflects the theoretical assumptions of the intervention being evaluated and the research question being addressed and that it has been applied using a patient-centred approach. Establishing the efficacy of the drug in this way is, of course, only part of establishing its utility – in terms of effectiveness and safety in the real world – and we will return to the problems associated with translating the findings of trials into everyday practice in section C. First, however, we will analyse the underlying assumptions that underpin the concept of 'outcomes' in such a trial and explore how these assumptions affect the measurement of change in evaluations of complex interventions, especially within the field of complementary and alternative medicine.

**Table 1:** OMERACT – best practice in disease-focused pharmaceutical trials

The first OMERACT conference on Outcome Measures in Rheumatoid Arthritis Clinical Trials [1] achieved agreement on a core set of outcomes measures to be used as a minimum in every clinical trial of rheumatoid arthritis (RA). These consist of acute phase reactants, disability, pain, patient global assessment, physician global assessment, swollen joint count, tender joint count, and radiographic studies of joints in any trial of 1 year or longer. Recently OMERACT has begun exploring the patient's perspectives of outcomes in RA [2,3]. Patients identified as important not just physical outcomes such as pain and disability, but also sleep disturbance, fatigue, "a general feeling of wellness" and "a return to normality".
Hughes et al [4] have investigated the extent to which this 'best practice' in outcome measurement for RA trials reflects the outcomes experienced after a complex intervention. Their work on the outcomes aimed for and experienced by a range of acupuncture practitioners treating people with RA indicates that even with the addition of the subjective patient-centred measures, the OMERACT set of outcome measures would miss many of the treatment effects that were identified, effects that were diverse, unpredictable and long-term.
1. Tugwell P, Boers M: **OMERACT conference on outcome measures in rheumatoid arthritis clinical trials: introduction**. *J Rheumatol *1993, **20: **528–530. 2. Kirwan J, Heiberg T, Hewlett S, Hughes R, Kvien T, Ahlmen M *et al*.: **Outcomes from the Patient Perspective Workshop at OMERACT 6**. *J Rheumatol *2003, **30: **868–872. 3. Carr A, Hewlett S, Hughes R, Mitchell H, Ryan S, Carr M *et al*.: **Rheumatology outcomes: the patient's perspective**. *J Rheumatol *2003, **30: **880–883. 4. Hughes JG, Goldbart J, Fairhurst E, Knowles K: **Exploring acupuncturists' perceptions of treating patients with rheumatoid arthritis**. *Complement Ther Med *2007, **15: **101–108.

#### Assumptions that underpin the concept of 'outcomes'

One result of the burgeoning field of 'outcomes' is that there is a tendency for 'outcomes' to be conceptualised as having some reality of their own, without a particular context and purpose. However, terms and concepts such as 'outcomes' are inevitably linked to underlying assumptions and meanings which may not be evident to users of the term. To continue our example of people with RA, we know that in addition to, or instead of, taking pharmaceutical treatments many such patients will also be using complementary therapies [31]. Consequently, there has been a call for evidence of the efficacy of complementary therapies that has largely been answered by designing trials which measure the same 'best practice' primary 'outcomes' as used in pharmaceutical trials. It is in this context that the underlying assumptions about 'outcomes' become important. Uncovering and critically analysing these assumptions enables us to free the concept of outcome, in the more general sense of 'consequence of', from the particular concept of 'outcomes' as used in efficacy or effectiveness research, dominated as it is by the RCT research paradigm. Through a process of multi-disciplinary group discussions during a residential workshop (see acknowledgements) we have identified the following assumptions as underpinning the concept of 'outcomes' as it is usually used within the context of clinical drug trials:

*1. An intervention is directed at preventing, moderating or curing one disease process*.

*2. The change in the disease process will be predictable and similar in its form for every patient (although variable in its degree)*.

*3. There is a linear cause and effect relationship between the intervention and the change in the disease process and the intervention is perceived as a separate entity not including the patient*.

*4. Context effects – such as the effects of the therapeutic relationship, the material and emotional aspects of the patients' life-worlds, changes in health beliefs and behaviours – are separate from the effect of the intervention under study*.

*5. Ideally, objective change indicators for the disease process are specified as the primary outcome. Subjective measures such as pain scores are only used as primary outcomes if there is no alternative, and in these situations high priority is given to developing new objective measures, by means of new technologies such as MRI scans and biochemical assays*.

*6. Subjective changes in health are a consequence of the change in the disease process, and are therefore measured as secondary outcomes. They do not usually contribute to the statistical outcome of a trial or evaluation*.

*7. The timeframe of the maximal response to treatment can be predicted by pharmacological and experimental evidence, so that the primary outcome is measured as a single endpoint. Longer term follow-up is an optional add-on to assess whether change is sustained*.

*8. It is often assumed that 'outcomes' relate primarily to changes that are being measured in randomised controlled trials*.

In the following section we will review the validity of each of these assumptions for evaluating complex interventions for people with long-term conditions. This will be followed by a discussion of how to move forward to an alternative conceptualisation of measuring change in these situations. First, however, we briefly address our use of the term 'complex intervention'.

#### Complex interventions

The term 'complex interventions' has been defined and used in a number of ways. The Medical Research Council's framework for the evaluation of complex interventions defines a complex intervention *as 'built up from a number of components, which may act both independently and inter-dependently' *and suggests that early phase research is necessary to define the 'active ingredients' [24,32]. Others have suggested that such interventions are better termed 'complicated' and that complexity, based on complexity theory, is represented by *'recursive causality (with reinforcing loops), disproportionate relationships (where at critical levels, a small change can make a big difference – a 'tipping point') and emergent outcomes' *[33] (pp29). A slightly different formulation is to describe a *complex systems approach*, which is promoted as making us *'consider the wider ramifications of intervening and to be aware of the interaction that occurs between components of the intervention as well as between the intervention and the context in which it is implemented' *[34]. In terms of health care, complex interventions are sometimes simply defined as *non-pharmaceutical *or as involving a high level of patient *participation*. In this paper we use the term 'complex intervention' to encompass complicated and complex health care interventions which are non-pharmaceutical and participative, such as the example of Tai Chi given above. Such interventions are the norm in many areas of health care such as complementary and alternative therapies, health promotion and rehabilitation.

#### The applicability of the above assumptions to evaluating complex interventions

Drawing on our own qualitative and quantitative work in the areas of patients' experiences of complex interventions, particularly complementary, alternative and integrative care, we will discuss to what extent each of the assumptions listed above holds true in the context of complex interventions.

*1. An intervention is directed at preventing, moderating or curing one disease process*.

AND

*2. The change in the disease process will be predictable and similar in its form for every patient (although variable in its degree)*.

Many complex interventions are focused on the whole individual person rather than on one disease process and in some cases this whole person approach is underpinned by an alternative therapeutic theory base, such as Chinese medicine theory or psychoanalytic theory. Although such interventions may take one symptom, or disease process, as a point of departure, the treatment process evolves to include the whole person and their history. In these situations there is no rational basis for a disease-focused outcome. Because the intervention is directed at each person as a unique individual, the nature of the changes, their timeframe and their social consequences will be unpredictable and individualised [13]. In addition these first two assumptions indicate that the patient is perceived as passive and accepting, whereas many complex interventions, especially in relation to people with long-term conditions, necessitate viewing the patient as an active participant in their own treatment strategy. We will return to this issue of people as active agents later, in section C, when we consider how to translate trial evidence into practice.

*3. There is a linear cause and effect relationship between the intervention and the change in the disease process and the intervention is perceived as a separate entity not including the patient*.

AND

*4. Context effects – such as the effects of the therapeutic relationship, the material and emotional aspects of the patients' life-worlds, changes in health beliefs and behaviours – are separate from the effect of the intervention under study*.

Many users, health care providers and researchers, are socialized into thinking that the intervention, understood as an autonomous technical intervention, is the determining factor in producing the particular outcomes. Whilst this may be largely true for the acute effects of powerful drugs, a linear cause and effect relationship is not consistent with complex interventions that are directed at the person as a whole being. In his classic text on holism [35], Smuts describes how the linear concept of cause and effect that is observed within mechanical structures undergoes a radical transformation when observed in the case of a holistic structure, such as an organism:

*'When an external cause acts on a whole, the resultant effect is not merely traceable to the cause, but has become transformed in the process. The whole seems to absorb and metabolise the external stimulus and to assimilate it into its own activity; and the resultant response is no longer the passive effect of the stimulus or cause, but appears as the activity of the whole' *[35]*(pp126)*.

More recent empirical research demonstrates that the effects of complex interventions are perceived, by both patients and therapists, to be related not only to the intervention itself but also to their communication and relationship, changes in patients' understanding of the meaning and cause of their symptoms, and the patients' own efforts and activities – factors that are sometimes termed 'context effects' [36]. Most importantly, these effects are not seen as separate from the specific intervention, but rather as the interplay between the intervention, the communication, the user's understanding of symptoms and the user's own efforts (actions) that develop in an ongoing process [13,16,30,37-40]. This process is also related to the patient's life situation, including the influence of social, cultural, economical and political factors [15,41]. In fact, the specific intervention may sometimes have less effect on the outcomes than the contextual and communicative aspects [7].

The experiences summarized in Table 2 illustrate this complexity and indicate that assumptions 3 and 4 are not reflected in these patients' experiences. The report given by Sussi in Table 2 illustrates how the reflexology treatment focused on the wider context of body-mind and activated Sussi's awareness and preventive behavior, thus combining the treatment prevention and health promotion. Self-regulating activities were set in motion, representing complex causal processes that led to positive processes and health-related change. Through the social interaction between Sussi and her therapist, the presenting symptom, headache, was transformed from a specific entity to a broader contextualized phenomenon.

**Table 2:** Data from a qualitative interview conducted in a research project on headache and reflexology treatment. [[Bibr B1]]

*"When the headaches started to disappear I told myself to calm down, because I expected them to return quickly – I actually did. But I just kept getting better....it was like being reborn....I had been spending a lot of time feeling unwell – time I should have spent with my family. It was really like being back in business. I actually think that it was my own mental process that made a change". In addition, the reflexologist has helped Sussi to get rid of her heartburn and bloated stomach – a problem that had previously required a lot of medicine – and has taught Sussi to press a key point in her ear if she got a bloated stomach. Sussi believes that the reflexology has also reduced her colds and sore throats, which had been frequent problems since childhood, and she no longer needed to use penicillin*.

*After the fifth treatment Sussi started to cut down a lot on her coffee intake: she says that her body now tells her what is good and what is bad for her. Whereas Sussi started with the feeling that the headaches were something she wanted to rip out, she now understands her headaches in another way and has a holistic understanding of her body*.

1. Brendstrup E, Launsø L. **Headache and reflexological treatment**. Copenhagen: The Council Concerning Alternative Treatment, National Board of Health; 1997.

The client was diagnosed by a medical specialist as having migraine at the start of the treatment project [1]

Consequently for complex interventions it is necessary to replace decontextualised, linear cause and effect models with more complex conceptual models that identify the assumptions that underlie the intervention as well as its process and context

*5. Ideally, objective change indicators for the disease process are specified as the primary outcome. Subjective measures such as pain scores are only used as primary outcomes if there is no alternative, and in these situations high priority is given to developing new objective measures, by means of new technologies such as MRI scans and biochemical assays*.

AND

*6. Subjective changes in health are a consequence of the change in the disease process, and are therefore measured as secondary outcomes. They do not usually contribute to the statistical outcome of a trial or evaluation*.

Patients seeking out non-pharmaceutical interventions such as complementary and alternative medicine often want help with multiple biomedical diagnoses. This makes the search for a single disease-focused primary outcome inappropriate. As illustrated by the data in Table 3, the outcomes for such patients may be very complex and they may be focused on other outcomes than mere health. The hierarchy of outcomes that results from prioritising objective outcomes over subjective ones, a hierarchy that is being promoted by the use of new scientific technologies such as MRI scans and biochemical assays, severely compromises the validity of outcome measurement for complex interventions. Measuring subjective outcomes but then excluding them from the main findings of the evaluation, is yet another example of the dominance of the medical gaze over the life-world of the patient [42,43]. We suggest that whilst it is helpful to differentiate between outcomes independent of the patient's awareness (blood tests, scanning etc.) and experience dependent on the patient's awareness (pain, gaining control etc) [8] this range of outcomes should be part of a complex model and understanding that illustrates that treatment effects are not purely, or sometimes not at all, the result of changes in disease processes.

**Table 3:** Main categories of 'outcomes' of complex interventions [1]

*1. Treatment results connected to body sensation* • *Physical effects *like physical symptoms diminishing or disappearing (pain, infections, constipation, tension, fatigue, etc.), strengthening of the immune system, etc. • *Mental effects *like removal of blockages, getting more energy, better sleep, increased quality of life, better general condition, feeling attended to/safeguarded. • *'Side gains' *like diminishing or disappearance of other physical symptoms than the ones that the user told the treatment provider about. • *Short term responses to the treatment *like a change in body odor, increased amount of faeces, change in the odour of the urine, head ache, old symptoms re-appearing. *• Long term responses *-

*2. Changes in awareness, understanding, insight* • *Increased bodily consiousness and bodily awareness *like being able to listen to and interpreting body signals. • *Changes in the knowledge and understanding of, and insight into *ones disease/symptoms, including putting into words other ways to understand disease than the biomedical understanding. • *Some sort of transformation*, understood as an individual, seeking, self integrating, and never ending health-related change process. • *Putting into words spiritual aspects and tools for working with spiritual aspects of life*. • *Greater awareness of one self in different social settings*.

3. *Changes in actions and development of new competences in the role as one's own 'disease manager' – especially important for people with chronic disease* • Develop a larger room of action and find ones own resources (drive). • Develop tools to handle life situations, including social activities. • Develop knowledge and tools to prevent symptoms and to promote health.

1. Launsø L: **Therapists' effect assumptions and users' own effort-when people with chronic diseases consult conventional and alternative therapists [in Danish with english abstract]**. *Tidsskrift for Forskning i Sygdom & Samfund [Journal of Research in Disease & Society] *2008, **9: **97–112.

*7. The timeframe of the maximal response to treatment can be predicted by pharmacological and experimental evidence, so that the primary outcome is measured at a single endpoint. Longer term follow-up is an optional add-on to assess whether change is sustained*.

This assumption, based like the others on the model of pharmaceutical research, is a poor fit for complex interventions, the effects of which are experienced by patients as part of longer chain of more or less interdependent effects – what we conceptualise as 'process' or 'change'. Effects may disappear, reappear and other changes may be experienced much later on [15,44,45]. In addition non-pharmaceutical interventions are themselves complex and relational and capable of adapting to individual patients changing wants and needs over time [13]. In this situation the use of 'outcomes' as an endpoint, results in such outcomes representing mere expressions of experiences conceptualised in a particular way at particular time in a particular place: when the patient had to fill in the questionnaire.

The importance of timeframe is accentuated in the case of people living with long-term illness. Research findings indicate that people go through different stages or phases of experience as they find their own individual way of coping and adapting to changing health and disability [46,47]. These 'illness careers' follow individualised paths which depend on personal characteristics, patterns of illness progression, whether there is experience of the illness amongst family and friends, and the wider cultural and material context of life. The effects of any interventions are intertwined with the effects of the associated biographical disruption [48] and attempts to rebuild personal and social identities [49-51]. Realistic aims may reflect a desire for safety, stability or a slowing of deterioration [52,53].

*8. It is often assumed that 'outcomes' relate primarily to changes that are being measured in randomised controlled trials*.

The position of randomised controlled trials at the apex of the evidence-based medicine approach to evaluation should not detract from the many other situations in which outcome measurement is important. These include early stage research that investigates the relationships between process, context and outcome over time; descriptive outcome studies; mixed method service evaluations; and practitioners seeking to improve their practice through case studies and audit. Not all these research methods require the measurement of 'before and after' primary quantitative outcomes and the reduction of change in health, wellbeing and health-related behaviours to single endpoints is unlikely to answer the questions posed. Being clear about the purpose of outcomes assessment, is a necessary pre-requisite for identifying indicators of change and how to measure, or observe, them.

### B. Addressing the Challenges of Measuring Change in Relation to Complex Interventions

In the previous section, we have demonstrated why and how the current concept of 'outcomes' and the way that it is commonly employed in clinical drug trials, is problematic in evaluating complex interventions, especially the use of complementary and alternative medicine by people with long-term health problems. We have used some examples from primary research to illustrate the challenges we face and how the choice of outcome affects the scope and level of knowledge that can be understood and described. The challenges that we have identified include the need to encompass and measure those changes that are intangible, rather than concrete; those that are individualised, rather than normative; those that are embedded in the context of the intervention, rather than clearly issuing from it; and those that are related to the discourses of contemporary society.

#### Using complex conceptual models

There are a number of different research frameworks, theoretical approaches and models that encompass different conceptualisations of outcomes. Frameworks include the recently updated MRC framework for evaluating complex interventions, which acknowledges the need to analyse multiple outcomes over longer time periods and to relate them to process [32] and the integrated quantitative and qualitative approach to developing and evaluating complex interventions exemplified by Bradley et al [54]. Other health service evaluators have used action research [55] and systems theory [41] approaches. In the field of public health the RE-AIM Model conceptualizes the public health impact of an intervention as a function of five factors: reach, efficacy, adoption, implementation and maintenance [56] and a classic model used in quality assurance in health care links outcomes to both structure and process [57].

In the field of complementary medicine research, a number of papers have developed conceptual models relating to outcomes from primary or secondary research [9,40,41] but these have generally not been based on a more generalisable theoretical framework. One exception to this has been the application of programme theory to evaluating complementary therapies and we will describe this work in more detail as an example of an alternative conceptualisation of outcomes. Further work in this area will allow for better understanding of the role of programme theory and other theoretical frameworks in evaluating a variety of complementary therapies in different populations and contexts.

#### Programme theory applied to complementary therapies

**Figure 2:**
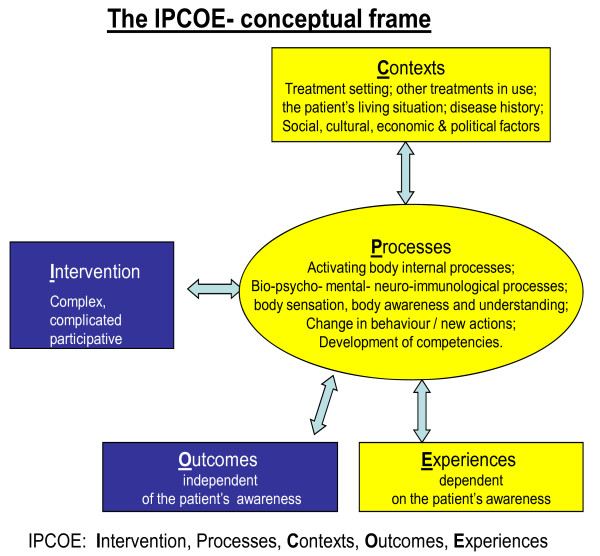
**IPCOE: a frame of reference for researching health-related change of complex interventions**.

Programme theory (intervention theory, logic modelling) has been developed to research and evaluate social policies, programmes and initiatives and it conceptualises outcome as linked to both mechanism and context [58-60]. It provides a tool to understand and explain what works for whom, in what circumstances, in what respects, and how. Used as a basis for constructing conceptual models of complex health care interventions, programme theory ensures that such models include not only the *intervention *and the *outcomes *but also explicitly represents the components and dynamic of the *process *and the social and cultural *context*s. The IPCOE framework depicted in Figure 2 is an example of such a model that has been developed in relation to team-based interventions involving conventional, complementary and alternative therapies for people with long-term health problems [8]. In this model 'outcomes' includes both 'outcomes independent of the patient's awareness' and 'experiences dependent on the patients awareness'.

The *intervention *is described in terms of its theoretical basis, as well as its components and dynamics, and this may include lay theories and assumptions as well as professional and therapy based theories and the wider social theories that come into play. In developing and applying the IPCOE framework, the first task has been to explore and make explicit the intervention theory (theories), including the practitioners' underlying assumptions about how an intervention is intended to work and what impacts this is expected to have. The *processes *in the IPCOE framework involve internal processes such as bio-psycho-mental-neuro-immunological processes, body sensation, body awareness and understanding; change in behaviour, testing out new actions and competencies and these processes can be perceived as a series of interrelated actions, changes, reactions or functions that happen over time as an individual moves from one state of health to another. Attending to process focuses on not only whether change occurred, but how the change occurred. *Context *is the set of circumstances within which the intervention and the processes take place, which may include the patient-provider relationship, treatment setting, other treatments in use, the patient's living situation and economic, social, cultural and political factors. The model highlights the interrelationships and feedback loops that occur over time such that the process is grounded in the social cultural and political contexts, unfolding over time and facilitating or inhibiting the outcomes. Research findings also indicate that the user's *learning *is an essential ingredient in their treatment trajectory [16,39,40,61].

The use of programme theory, using tools based on the IPCOE framework, is proving valuable in an investigation of whether collaboration between conventional and alternative practitioners may optimize treatment results for people who have multiple sclerosis [8]. In the first stage of the project each therapist was asked to fill in the framework for their respective treatment models outlining typical interventions, the assumed mechanisms, expected outcomes, and relevant contextual factors that may influence the outcome positively or negatively. These were then shared between practitioners and discussed at practitioner-researcher seminars, a process that demonstrated and made explicit the significant differences between conventional and alternative practitioners and led to self-reflection and improved understanding of how the team could work together.

#### The 'outcomes' concept in programme theory

In a framework such as that depicted in Figure 2, it is clear that there is not a linear cause-and -effect relationship between intervention and outcome, but rather that health-related change results from the interaction between intervention, process and context over time. Thus the interventions achieve their effects via the active input of the individual internal processes and contexts [59]. In this framework both the intervention and the patient are defined as causal factors, because the result of the treatment is dependent on the resources of the patient – such as the body's ability to heal itself – and the impact of the patient's situation. In addition, rather than depicting outcomes as objective and subjective, the framework includes both 'outcomes independent of the patient's awareness' and 'experiences dependent on the patient's awareness' and accords them equal significance. Using the concept of experiences, as against subjective outcomes, highlights the dynamic and relational aspect of this phenomenon and its connection with learning. Learning and experience interact, based on the treatment intervention, communication between practitioner and user, the user's efforts and actions and the user's social contexts [16,39,40,61]. Such experiences will include categories such as 'new holistic understandings'[40], increased self-awareness [9], and 'learning new things' [62] found in other studies of complementary medicine. Using such a model based on programme theory results in a wide range of health-related changes and a list of potential changes is provided in Table 3. This list, and the inclusion of the category '*Changes in actions and the development of new competences in the role as one's own 'disease manager' *is based on research with people with a variety of chronic health problems who accessed complementary and alternative treatment [16,63]. Similar categories of change have been identified by other researchers into complementary and integrative health care [9,13,30,44,45,64,65].

### C: Applying the Benefits of Complex Conceptual Models to Implementing Trial Results

Although we have indicated that the assumptions underlying the measurement of 'outcomes' in clinical drug trials are generally appropriate for evaluating pharmaceuticals, there is a particular aspect of these research programmes which would benefit from a greater awareness of the various interpretations and alternative models of outcomes. This aspect is the implementation of the findings of clinical drug trials into routine practice – an aspect that is causing increasing concern within the evidence-based medicine movement and in health service policy. For example, research shows that despite all the new antihypertensive drugs, the blood pressure of the majority of hypertensive patients is inadequately controlled [66] and that only about 50% of drugs that are prescribed are taken correctly [67]. This suggests that, even for pharmaceutical interventions, their use in the real world constitutes a complex intervention in which the underlying assumptions, beliefs and everyday contexts of both patients and providers play important and inter-related roles. Developing conceptual models of these complexities will help to encourage awareness of different interpretations of 'outcomes' that take into account a wider range of issues that are affecting patient and provider behaviour. For example, a synthesis of the qualitative studies of the lay experience of medicine taking found that the main reason why people do not take their medicines as prescribed is because of a lay resistance to taking medicines [68]. The synthesis depicts the complex nature of this resistance and the associated behaviours as a diagrammatic 'model of medicine taking' which can be used to improve concordance and adherence.

## Summary

This critical analysis of the concept and term 'outcomes', as commonly used in clinical drug trial research, has uncovered the underlying assumptions on which it has, necessarily, been constructed. This analysis indicates that in this context 'outcomes' are often conceptualised as disease-focused endpoints of a linear cause and effect intervention, which are ideally measured in terms of a single objective primary outcome at a fixed time-point. Context effects, which include aspects of the process of the intervention and the wider context of peoples' lives, are viewed as separate entities which are best removed from the evaluation process by means of a randomised controlled trial design. Our analysis indicates that whilst this concept of outcome may be appropriate for measuring the short-term efficacy of pharmaceutical interventions, it is inappropriate for most complex interventions. Even within pharmaceutical based research, this concept of 'outcomes' is insufficient for measuring the effectiveness of interventions in the real world, or for understanding and intervening in the implementation of clinical trial findings.

The use of complex conceptual models, such as those based on programme theory, are a basis for understanding the complexity of the experience of health-care interventions within the wider social and cultural context of peoples' lives. They are an alternative basis on which to understand and evaluate the changes in health and wellbeing associated with many complementary and alternative medicine interventions and to understand the role of various factors in promoting positive changes. Our own work with people with long-term conditions who use complementary therapies illustrates that models built on the findings of in-depth qualitative research provide a basis on which to design evaluations and develop indicators of change. These indicators of change need to include aspects of process, such as new meanings and understanding, as well as longer term changes in health, wellbeing and health-related competences and behaviours. These models lead to a different interpretation of 'outcomes' which encompasses the interactions and learning that constitutes the treatment experience over time, and views the patient as an active agent who will interact with an intervention in ways that produce individualised changes. Current and future work in this field will improve our ability to measure aspects of these complex changes with quantitative instruments and questionnaires, and to combine this with qualitative methods in mixed-method evaluations.

## Competing interests

The authors declare that they have no competing interests.

## Authors' contributions

All authors conceived and discussed the critical analysis and contributed to drafting and writing the paper. CP took the lead in drafting the final manuscript. All authors read and approved the final manuscript.

## Pre-publication history

The pre-publication history for this paper can be accessed here:



## References

[B1] Ostermann T, Bussing A, Beer AM, Matthiessen PF (2005). The Herdecke Questionnaire on Quality of Life (HLQ): validation of factorial structure and development of a short form within a naturopathy treated in-patient collective. Health Qual Life Outcomes.

[B2] Long AF, Mercer G, Hughes K (2000). Developing a tool to measure holistic practice: a missing dimension in outcomes measurement within complementary therapies. Complement Ther Med.

[B3] Paterson C (1996). Measuring outcomes in primary care: a patient generated measure, MYMOP, compared with the SF-36 health survey. BMJ.

[B4] Campbell R, Quilty B, Dieppe P (2003). Discrepancies between patients' assessments of outcome: qualitative study nested within a randomised controlled trial. BMJ.

[B5] Carr AJ, Higginson IJ (2001). Are quality of life measures patient centred?. BMJ.

[B6] Edwards SG, Playford ED, Hobart JC, Thompson AJ (2002). Comparison of physician outcome measures and patients' perception of benefits of inpatient neurorehabilitation. BMJ.

[B7] Kaptchuk TJ, Kelley JM, Conboy LA, Davis RB, Kerr CE, Jacobson EE, Kirsch I, Schyner RN, Nam BH, Nguyen LT, Park M, Rivers AL, McManus C, Kokkotou E, Drossman DA, Goldman P, Lembo AJ (2008). Components of placebo effect: randomised controlled trial in patients with irritable bowel syndrome. BMJ.

[B8] Launsø L, Skovgaard L (2008). The IMCO scheme as a tool in developing team-based treatment for people with multiple sclerosis. J Altern Complement Med.

[B9] Long AF (2002). Outcome measurement in complementary and alternative medicine: unpicking the effects. J Altern Complement Med.

[B10] Paterson C, Thomas K, Manasse A, Cooke H, Peace G (2007). Measure Yourself Concerns and Wellbeing (MYCaW): an individualised questionnaire for evaluating outcome in cancer support care that includes complementary therapies. Complement Ther Med.

[B11] Verhoef MJ, Balneaves LG, Boon HS, Vroegindewey A (2005). Reasons for and characteristics associated with complementary and alternative medicine use among adult cancer patients: a systematic review. Integr Cancer Ther.

[B12] Verhoef MJ, Vanderheyden LC, Fonnebo V (2006). A whole systems research approach to cancer care: why do we need it and how do we get started?. Integr Cancer Ther.

[B13] Baarts C, Pedersen IK (2009). Derivative benefits-exploring the body through alternative and complementary medicine. Sociol Health Ill.

[B14] Fitzpatrick RM, Hopkins AP, Harvard-Watts O (1983). Social dimensions of healing: a longitudinal study of outcomes of medical management of headaches. Soc Sci Med.

[B15] Glik DC, Kelner M, Wellman B (2000). Incorporating symbolic, experiential and social realities into effectiveness research on CAM. Complementary and Alternative Medicine: Challenge and Change.

[B16] Launsø L, Henningsen I, Rieper J, Brender H, Sando F, Hvenegaard A (2007). Expectations and effectiveness of medical treatment and classical homeopathic treatment for patients with hypersensitivity illnesses – one year prospective study. Homeopathy.

[B17] Mulkins A, Verhoef M, Eng J, Findlay B, Ramsum D (2003). Evaluation of the Tzu Chi Institute for Complementary and Alternative Medicine's Integrative Care Program. J Altern Complement Med.

[B18] Trauer T (1998). Issues in the assessment of outcome in mental health. Aust N Z J Psychiatry.

[B19] Holmes J (2002). All you need is cognitive behaviour therapy?. BMJ.

[B20] Launsø L, Rieper J (2005). General practitioners and classical homeopaths treatment models for asthma and allergy. Homeopathy.

[B21] Mason S, Tovey P, Long AF (2002). Evaluating complementary medicine: methodological challenges of randomised controlled trials. BMJ.

[B22] Power R, Budd S, Sharma U (1994). 'Only nature heals': a discussion of therapeutic responsibility from a naturopathic point of view. The Healing Bond.

[B23] Aickin M (2007). The importance of early phase research. J Altern Complement Med.

[B24] Campbell M, Fitzpatrick R, Haines A, Kinmonth AL, Sandercock P, Spiegelhalter D, Tyrer P (2000). Framework for design and evaluation of complex interventions to improve health. BMJ.

[B25] Hawe P, Shiell A, Riley T (2004). Complex interventions: how "out of control" can a randomised controlled trial be?. BMJ.

[B26] Kotaska A (2004). Inappropriate use of randomised trials to evaluate complex phenomena: case study of vaginal breech delivery. BMJ.

[B27] Fønnebo V, Grimsgaard S, Walach H, Ritenbaugh C, Norheim AJ, MacPherson H, Lewith G, Launso L, Koithan M, Falkenberg T, Boon H, Aickin M (2007). Researching complementary and alternative treatments – the gatekeepers are not at home. BMC Med Res Methodol.

[B28] Paterson C, Dieppe P (2005). Characteristic and incidental (placebo) effects in complex interventions such as acupuncture. BMJ.

[B29] Verhoef MJ, Lewith G, Ritenbaugh C, Boon H, Fleishman S, Leis A (2005). Complementary and alternative medicine whole systems research: beyond identification of inadequacies of the RCT. Complement Ther Med.

[B30] Verhoef MJ, Mulkins A, Boon H (2005). Integrative health care: how can we determine whether patients benefit?. J Altern Complement Med.

[B31] Fautrel B, Adam V, St Pierre Y, Joseph L, Clarke AE, Penrod JR (2002). Use of complementary and alternative therapies by patients self-reporting arthritis or rheumatism: results from a nationwide Canadian survey. J Rheumatol.

[B32] Craig P, Dieppe P, Macintyre S, Michie S, Nazareth I, Petticrew M (2008). Developing and evaluating complex interventions: the new Medical Research Council guidance. BMJ.

[B33] Rogers PJ (2008). Using programme theory to evaluate complicated and complex aspects of interventions. Evaluation.

[B34] Shiell A, Hawe P, Gold L (2008). Complex interventions or complex systems? Implications for health economic evaluation. BMJ.

[B35] Smuts JC (1927). Holism and Evolution.

[B36] Di Blasi Z, Harkness E, Ernst E, Georgiou A, Kleijnen J (2001). Influence of context effects on health outcomes: a systematic review. Lancet.

[B37] Hughes JG, Goldbart J, Fairhurst E, Knowles K (2007). Exploring acupuncturists' perceptions of treating patients with rheumatoid arthritis. Complement Ther Med.

[B38] Johannesse H, Cant S, Sharma U (1996). Individualized knowledge: reflexologists, biopaths and kinesiologists in Denmark. Complementary and Alternative Medicines: Knowledge in Practice.

[B39] Luff D, Thomas KJ (2000). 'Getting somewhere', feeling cared for: patients' perspectives on complementary therapies in the NHS. Complement Ther Med.

[B40] Paterson C, Britten N (2004). Acupuncture as a complex intervention: a holistic model. J Altern Complement Med.

[B41] Paterson C, Vindigni D, Polus B, Browell T, Edgecombe G (2008). Evaluating a massage therapy training and treatment programme in a remote Aboriginal community. Complement Ther Clin Pract.

[B42] Mishler E (2007). The Discourse of Medicine: Dialectics of Medical Interviews.

[B43] Paterson C (2008). The colonization of the lifeworld of acupuncture: the SAR conference. J Altern Complement Med.

[B44] Koithan M, Verhoef M, Bell IR, White M, Mulkins A, Ritenbaugh C (2007). The process of whole person healing: "unstuckness" and beyond. J Altern Complement Med.

[B45] Paterson C, Britten N (2003). Acupuncture for people with chronic illness: combining qualitative and quantitative outcome assessment. J Altern Complement Med.

[B46] Avis M, Bond M, Antony A (1997). Questioning patient satisfaction: an empirical investigation in two outpatient clinics. Soc Sci Med.

[B47] Bury M (1991). The sociology of chronic illness: a review of research and prospects. Sociol Health Ill.

[B48] Bury M (1988). Chronic illness as biographical disruptions. Sociol Health Ill.

[B49] Charmaz K, Roth JA, Conrad P (1987). Struggling for self: identity levels among the chronically ill. The Experience and Management of Chronic Illness.

[B50] Kelly-Powell ML (1997). Personalizing choices: patients' experiences with making treatment decisions. Res Nurs Health.

[B51] Williams G (1984). The genesis of chronic illness: narrative re-construction. Sociol Health Ill.

[B52] Cartwright T, Torr R (2005). Making sense of illness: the experiences of users of complementary medicine. J Health Psychol.

[B53] Low J (2004). Managing safety and risk: the experiences of people with Parkinson's disease who use alternative and complementary therapies. Health (London).

[B54] Bradley F, Wiles R, Kinmonth AL, Mant D, Gantley M (1999). Development and evaluation of complex interventions in health services research: case study of the Southampton heart integrated care project (SHIP). The SHIP Collaborative Group. BMJ.

[B55] Greenhalgh T, Collard A, Begum N (2005). Sharing stories: complex intervention for diabetes education in minority ethnic groups who do not speak English. BMJ.

[B56] Glasgow RE, Vogt TM, Boles SM (1999). Evaluating the public health impact of health promotion interventions: the RE-AIM framework. Am J Public Health.

[B57] Donabedian A (2003). An Introduction to Quality Assurance in Health Care.

[B58] Pawson R, Tilley N (1997). Realistic Evaluation.

[B59] Pawson R, Greenhalgh T, Harvey G, Walshe K (2005). Realist review – a new method of systematic review designed for complex policy interventions. J Health Serv Res Policy.

[B60] McLaughlin JA, Jordan G, Wholey J, Hatry H, Newcomer KE (2004). Using logic models. Handbook of Practice Program Evaluation.

[B61] Cassidy C, Emad M, Cassidy CM (2002). What patients say about Chinese medicine. Contemporary Chinese Medicine and Acupuncture.

[B62] Cassidy CM (1998). Chinese medicine users in the United States. Part II: Preferred aspects of care. J Altern Complement Med.

[B63] Launsø L (2008). Therapists' effect assumptions and users' own effort-when people with chronic diseases consult conventional and alternative therapists [in Danish with English abstract]. Tidsskrift for Forskning i Sygdom & Samfund [Journal of Research in Disease & Society].

[B64] Miller WL, Crabtree BF, Duffy MB, Epstein RM, Stange KC (2003). Research guidelines for assessing the impact of healing relationships in clinical medicine. Altern Ther Health Med.

[B65] Mulkins AL, Verhoef MJ (2004). Supporting the transformative process: experiences of cancer patients receiving integrative care. Integr Cancer Ther.

[B66] Hajjar I, Kotchen TA (2003). Trends in prevalence, awareness, treatment, and control of hypertension in the United States, 1988–2000. JAMA.

[B67] World Health Organization (2003). Adherence to long-term therapies: evidence for action Geneva.

[B68] Pound P, Britten N, Morgan M, Yardley L, Pope C, Daker-White G, Campbell R (2005). Resisting medicines: a synthesis of qualitative studies of medicine taking. Soc Sci Med.

